# Suppression of IRE1*α* Attenuated the Fatty Degeneration in Parenteral Nutrition-Related Liver Disease (PNALD) Cell Model

**DOI:** 10.1155/2020/7517540

**Published:** 2020-02-03

**Authors:** Ningxun Cui, Mingling Cui, Jie Li, Xueping Zhu, Xiaoli Zhu

**Affiliations:** ^1^Department of Neonatology, Children's Hospital of Soochow University, Suzhou, Jiangsu 215025, China; ^2^Department of Intervention, The First Affiliated Hospital of Soochow University, Suzhou, Jiangsu 215006, China

## Abstract

**Aims:**

To model the parenteral nutrition-associated liver disease (PNALD) in rat normal hepatocytes BRL and investigate the role of endoplasmic reticulum stress- (ERS-) related IRE1*α* signal in the process of PNALD.

**Methods:**

The BRL cells were treated with different concentrations of soybean oil emulsion (SO) to induce hepatocyte fatty degeneration. The PNALD cell disease model was further confirmed by analysis of Oil Red O staining and biochemical parameters. Next, the IRE1*α* signal in the process of PNALD. *α* signal in the process of PNALD. *α* signal in the process of PNALD. *α* signal in the process of PNALD.

**Results:**

The results of Oil Red O staining indicated that the PNALD was successfully established in BRL cells and the CCK-8 data indicated which 0.6% that SO was further applied to the experiment owing to its better induction of PNALD and less toxicity to the cells. Besides, the value of biochemical parameters (TBIL, DBIL, ALT, and AST) was also elevated in the SO group compared with the NG group. After knockdown of IRE1*α* signal in the process of PNALD. *α* signal in the process of PNALD.

**Conclusion:**

IRE1*α* was induced in PNALD cell model and suppression of IRE1*α* resulted in reduced steatosis in this cell disease model. Taken together, our data suggested that the IRE1*α* pathway may be involved in the development of PNALD.*α* signal in the process of PNALD. *α* signal in the process of PNALD. *α* signal in the process of PNALD.

## 1. Introduction

Parenteral nutrition (PN) has revolutionized the lifestyle of the neonates with growth defect caused by intestinal dysfunction [1]. The first case of long-term parenteral nutrition in newborn was reported in the USA last century [2]. Since PN seems to be the best effective therapy on these defects, the number of the patients, both young and aged, depending on PN for survival was growing yearly [3]. Unfortunately, long-term application of PN could develop into serious diseases, such as PN-associated liver disease (PNALD), which could lead to a high incidence of morbidity and mortality [4–6]. According to the report, about 50% to 66% of kids receiving long-term PN finally developed into PNALD [7]. Although some recognized risk factors, including premature birth, long-term preservation of PN, low-quality of newborns, and the fat composition [8–10], have been attributed to PNALD, the definitive and specific etiology and pathogenesis still remains uncertain.

In eukaryotic cells, the endoplasmic reticulum (ER) is essential for the folding and trafficking of proteins that enter the secretory pathway. ER orchestrates the synthesis, folding, and transport of at least one-third of the proteins in eukaryotic cells. Because of the high active protein synthetic activity in the hepatocytes, the abundant copy and precise regulation as well as organization of ER were required. Previous studies demonstrated that dysfunction of ER, caused by ER stress, may contribute to human diseases including liver disease [11, 12]. During the process of ER stress, ER homeostasis will collapse and an unfolded protein response (UPR) get initiated [13]. UPR was regulated by three transducers, inositol-requiring enzyme 1 (IRE1), protein kinase R-like ER kinase (PERK), and activating transcription factor 6 (ATF6), in the ER network [14]. Commonly, they bind to glucose-regulated protein 78 (GRP-78) on the ER membrane to promote protein folding and prevent protein aggregation using adenosine triphosphate (ATP). Among the ER-resident chaperones, GRP-78 is the master initiator of UPR signaling [15]. Recently, ER stress was reported in the pathogenesis of nonalcoholic fatty liver disease (NAFLD), hepatocellular carcinoma caused by hepatitis B virus, intestinal failure-associated liver disease (IFALD), and alcoholic liver disease (ALD) [16–19]. Zhang et al. reported that ER stress was positively correlated with PNALD and, with activation of autophagy by rapamycin, could protect against PNALD via suppressing ROS-induced ER stress [20]. Our previous study also demonstrated that soybean oil-based lipid emulsions could induce significant ER and mitochondrial damage, ultimately resulting in ER stress in primary rabbit hepatocytes [21, 22]. Thus, the previous studies suggested that ER stress may be involved in the pathogenesis and development of PNALD.

In this research, rat normal hepatocytes were subjected to soybean oil-based lipid emulsion (SO) treatment to model PNALD. Besides, IRE1*α* was suppressed by specific shRNA in hepatocytes to investigate the role of ER stress in PNALD model.

## 2. Materials and Methods

### 2.1. Rat Normal Hepatocytes

The rat normal hepatocytes (BRL) were kindly provided by Stem Cell Bank, Chinese Academy of Sciences, China. For routine maintenance, the BRL cells were cultured in DMEM (Thermo Fisher, USA) medium containing 10% FBS (Gibco, US) and 1% penicillin-streptomycin (Thermo Fisher, USA) at 37°C in a 5% CO_2_ incubator. The medium was changed daily until required spilt.

### 2.2. Lentivirus Production

The shRNAs targeting IRE1*α* were synthesized by Genewiz and annealed as previously reported. The shRNA product was then ligated into the backbone vector. The virus packaging was processed with psPAX2 (Addgen #12260) and pMD2.G (Addgen #12260) into the 293NT cells. After ultracentrifugation, the virus was collected and resuspended in the medium [23].

### 2.3. Establishment of IRE1*α*-Suppressed BRL Cell Lines

According to the IRE1*α* protein expression, the shRNA-#2 was used to transduced the BRL cells. After infection of lentivirus, the cells were selected with puromycin for 3 weeks. Finally, the BRL cells were all GFP positive and the expression of IRE1*α* was hardly detected, indicating that the IRE1*α*-suppressed BRL cell line was successfully established named shIRE1*α*. Simultaneously, the negative control shRNA was also induced into the BRL cells termed shControl.

### 2.4. Modeling of PNALD in BRL Cells

According to the previous report [21], the procedure was mildly modified below. The BRL hepatocytes were treated with different concentrations of SO (0.2%, 0.4%, 0.6%, 1%, and 2% diluted in DMEM from 20% SO). The 0.6% concentrations was used for all follow-up experiments. The SO was obtained from Sino-Swed Pharmaceutical, China.

### 2.5. CCK-8 Assay

BRL hepatocytes were seeded into 96-well plates at a density of 2 × 10^4^ cells per well and treated as previously described. Cells were then cultured for 24 h and CCK-8 analysis was performed on them according to the instruction. The absorbance of the formazan derivative was measured at 450 nm using a microplate reader (DNM-9602; Shengke, Shanghai, China). All measurements were performed in triplicate, and all experiments were repeated three times.

### 2.6. Oil Red O Staining

The lipid droplet accumulation was detected by Oil Red O staining. Briefly, after cells were treated with SO as indicated, the cells were fixed in 10% (*v*/*v*) formaldehyde for 10 min and then stained with Oil Red O solution (0.5% in isopropanol, *w*/*v*) for 15 min. Following extensive washes with distilled water, cells were stained with hematoxylin for 10 min. The representative images were taken under a light microscope.

### 2.7. Biochemical Analysis

Culture media from the experimental groups were collected. Various biochemical parameters, including total bilirubin (TBIL), direct bilirubin (DBIL), alanine aminotransferase (ALT), and aspartate aminotransferase (AST), were measured from each sample using an automatic biochemical analyzer (LXTM20, Beckman, USA).

### 2.8. Western Blotting

The cells were treated as indicated and then washed twice with PBS, followed by lysed in radio immunoprecipitation assay (RIPA) buffer containing 50 mM Tris-HCl (pH 7.5), 150 mM NaCl, 1% sodium deoxycholate, 1% Triton X-100, 5 nM ethylenediaminetetraacetic acid, 0.1% sodium dodecyl sulfate (SDS), and complete proteinase inhibitor (Roche, Mannheim, Germany). After incubation on ice for 30 min, lysates were briefly sonicated and nonsoluble cell debris was removed through centrifugation at 4°C. Protein lysates in SDS loading buffer were heated for 10 min at 95°C and then electrophoresed in SDS-PAGE gel. Protein samples were transferred onto polyvinylidene fluoride (PVDF) membranes and probed with indicated primary antibodies for 12 h at 4°C after being blocked in 5% nonfat dry milk. The PVDF membrane was incubated with appropriate secondary antibodies conjugated to horseradish peroxidase for 1 h at 25°C. Finally, protein was detected using chemiluminescent horseradish peroxidase substrates with Molecular Imager Gel Doc XR+ System (Bio-Rad).

### 2.9. Statistical Analysis

All values are expressed as mean ± SD from replicates, and Student's *t* test (paired) was performed using GraphPad Prism software. A *P* value of less than 0.05 was considered statistically significant.

## 3. Results

### 3.1. Modeling of PNALD in BRL Cells

**Figure 1:**
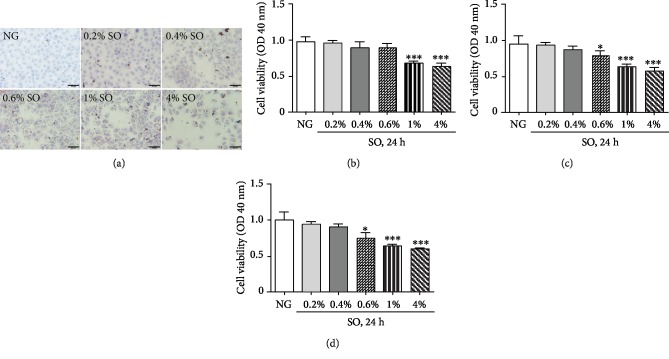
Cell modeling of PNALD *in vitro*. (a) Rat normal hepatocytes BRL were treated with different concentrations of soybean oil emulsion (SO). The degree of steatosis in BRL cells was determined by Oil Red O staining. Scar bar: 50 *μ*m. (b) BRL cell viability was analyzed by the method of CCK-8 after treated with SO. NG: not given (BRL cells untreated with SO). The data was presented as mean ± SD^∗∗∗^*P* < 0.001. (c) BRL cell viability was analyzed by the method of CCK-8 after treatment with SO for 48 h. NG: not given (BRL cells untreated with SO). The data was presented as mean ± SD, ^∗^*P* < 0.05, ^∗∗∗^*P* < 0.001. (d) BRL cell viability was analyzed by the method of CCK-8 after treatment with SO for 72 h. NG: not given (BRL cells untreated with SO). The data was presented as mean ± SD, ^∗^*P* < 0.05, ^∗∗∗^*P* < 0.001.

As presented in Figure 1(a), when the cells treated with SO for 24 h from 0.1% to 2%, the extension of Oil Red O staining was increased in a dose-dependent manner, indicating that the lipid droplet accumulation was acquired by administration of SO. Next, to get an optimal SO concentration from 24 to 72 h, cell cytotoxicity was also analyzed by the method of CCK-8. As shown in Figures 1(b)–1(d), when the cells are treated with SO under the concentration of 0.6%, nonsignificant cytotoxicity was detected, while above this concentration, the BRL cells exhibited serious cytotoxicity. Therefore, 0.6% SO was applied to further experiments for its greater lipid droplet accumulation and little cytotoxicity.

**Figure 2:**
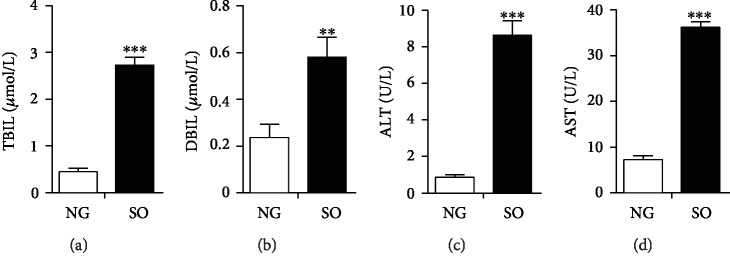
Biochemical parameters of BRL cells after treatment with SO. (a) The content of total bilirubin (TBIL) in cell medium. (b) The content of direct bilirubin (DBIL) in cell medium. (c) The content of alanine aminotransferase (ALT) in cell medium. (d) The content of aspartate aminotransferase (AST) in cell medium. NG: not given (BRL cells untreated with SO). The data was presented as mean ± SD, ^∗∗^*P* < 0.01, ^∗∗∗^*P* < 0.001.

After treated with SO for 24 h, the function of hepatocytes was analyzed by several biochemical parameters, including TBIL, DBIL, ALT, and AST. When the liver damage occurred, it could result in an accumulation of TBIL, DBIL, ALT, and AST. Surprisingly, all the values of these factors were consistently elevated (Figures 2(a)–2(d)). Taken together, these data suggested 0.6% SO could induce PANLD in BRL cells, indicating that the BRL-PANLD cell disease model was successfully established.

### 3.2. Generation of IRE1*α*-Suppressed BRL Cells

**Figure 3:**
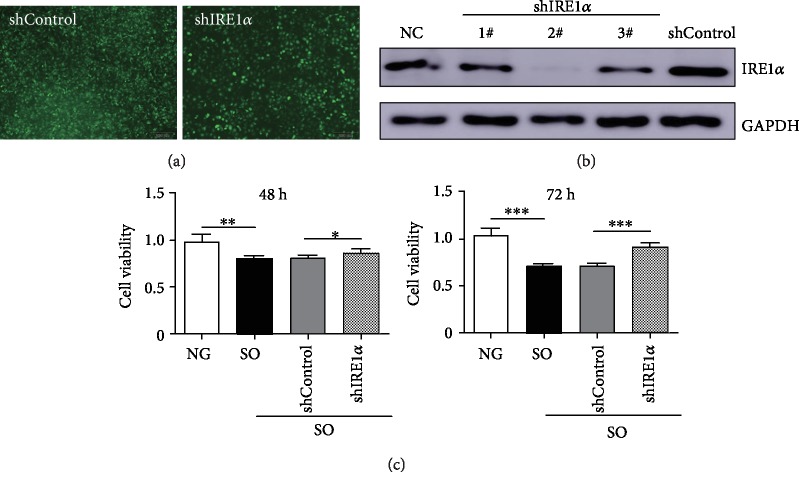
Knockdown of IRE1*α* in BRL cells. (a) The representative images of BRL cells transfected with indicated lentivirus. shControl indicated the negative control shRNA and the shIRE1*α* was the shRNA targeted shIRE1*α*. Scar bar: 50 *μ*m. (b) The protein expression was detected by western blotting. GAPDH was loaded as an internal control. NC indicated BRL cells not infected with any virus. (c) Cell viability was analyzed by the method of CCK-8 after knockdown of IRE1*α* when exposed to SO for 48 h and 72 h, respectively. NG: not given (BRL cells untreated with SO). The data was presented as mean ± SD, ^∗^*P* < 0.05, ^∗∗^*P* < 0.01, ^∗∗∗^*P* < 0.001.

The BRL cells were transduced with lentivirus carrying shControl or shIRE1*α*. After selection with puromycin (0.8 *μ*g/mL) for 3 weeks, almost all the cells were positive for GFP (Figure 3(a)). The expression of IRE1*α* was examined by the method of western blotting, only shIRE1*α*-2# acquired efficient suppression of the target (Figure 3(b)). Thus, the shIRE1*α*-2# cell line was subjected to further study. Besides, the SO-induced cytotoxicity was also analyzed after knockdown of IRE1*α*, and the data obtained from CCK-8 analysis suggested silence of IRE1*α* that could protect cells from cytotoxicity when treated with SO for long term (Figure 3(c)).

### 3.3. IRE1*α* Suppression Attenuated the Fatty Degeneration in BRL Cells

**Figure 4:**
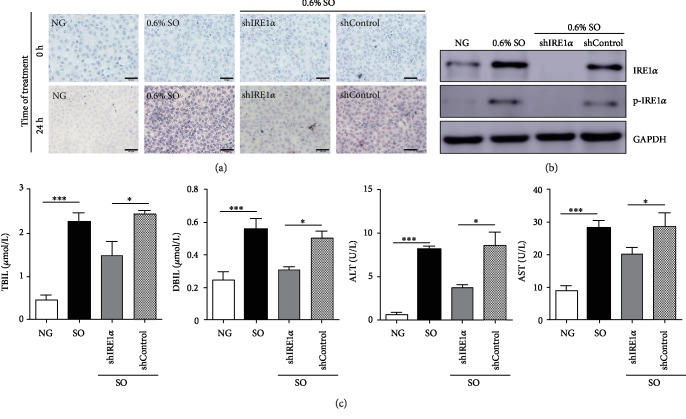
IRE1*α* suppression attenuated the steatosis in BRL cells. (a) The representative images of Oil Red O staining in BRL cells in different groups as indicated. Scar bar: 50 *μ*m. (b) The protein expression was detected by western blotting. GAPDH was loaded as an internal control. NC indicated BRL cells not infected with any virus. (c) Biochemical parameters (TBIL, DBIL, ALT, and AST) of BRL cells in different groups as indicated. NG: not given (BRL cells untreated with SO). The data was presented as mean ± SD, ^∗^*P* < 0.05, ^∗∗∗^*P* < 0.001.

Finally, to test whether ER stress-related IRE1*α* signal was involved in the development of PANLD *in vitro*, the cells were treated with 0.6% SO as indicated; after staining with Oil Red O, the cell morphology was normal and little lipid droplet accumulation was seen when treated for 0 h. Interestingly, when treated for 24 h, the lipid droplet accumulation was obvious both in the 0.6% SO group and 0.6% SO-shControl group, but less lipid droplets are accumulated in the 0.6% SO-shIRE1*α* group, indicating that suppression of IRE1*α* could protect against PANLD (Figure 4(a)). Besides, the expression of IRE1*α* was also determined after treatment. When treated with 0.6% SO, the expression of IRE1*α* was dramatically increased while little expression was detected in the group of shIRE1*α*. When ER stress was inhibited by knockdown of IRE1*α*, the expression of IRE1*α* was only accumulated in the cells treated with SO and SO-shControl group (Figure 4(b)). Besides, to further assess the function of hepatocytes, the biochemical parameters, including TBIL, DBIL, ALT, and AST, were analyzed. When treated with SO, compared with the NG group, all the values were increased in the SO and SO-shControl groups. Once IRE1*α* is inhibited although treated with SO, the values of TBIL, DBIL, ALT, and AST were also decreased compared with the shControl group. Taken together, these data suggested that the inhibition of IRE1*α* could attenuate the fatty degeneration of PANLD in BRL disease cell model.

## 4. Discussion

In this study, we successfully established rat hepatocytes PNALD cell disease model using soybean oil-based lipid emulsion system. Besides, the changes in several biochemical parameters that reflect hepatic function, which include LD accumulation and ER stress-related signals, were compared between the groups. Furthermore, the IRE1*α*-suppressed BRL cell line was established to investigate its role in the development of PNALD. The results demonstrated that when IRE1*α* is inhibited, the development of PNALD was decreased, suggesting that blocking of ER stress pathway could benefit for liver function when suffering PNALD. Our data suggested that ER stress-related signal was highly activated in PNALD models, which was consistent with the previous reports [17, 24, 25]. The previous clinical trials also demonstrated that the dosage and timespan of lipid emulsion application were positively correlated with the incidence of PNALD [26]. In our data, when treated with high concentration (above 1%) of SO for long term (48 h or 72 h), the lipid droplet accumulation was much higher than the lower group (under 0.6%), this was consistent with the clinical evidence.

The endoplasmic reticulum (ER) governs the proper folding and posttranslational modification of secreted and transmembrane proteins. Diverse physiological and pathological conditions can provoke the accumulation of misfolded proteins within this organelle. These, in turn, can induce ER stress and activate the unfolded protein response (UPR) [27]. Recently, emerging evidence indicates that IRE1*α* signaling can also control UPR-independent cellular pathways, influencing processes such as hepatic lipogenesis, angiogenesis, atherosclerosis, arthritis, and antitumor immunity [28–32]. Whether this conserved signal pathway was involved in the pathogenesis of PNALD was still uncertain. In this study, we first found that IRE1*α* was highly expressed in SO-treated BRL cells. When IRE1*α* is inhibited, the fatty degeneration was also suppressed. All these data suggested that IRE1*α* may be involved in the development of PNALD. But the underlying molecular mechanism still remains to be investigated.

Collectively, soybean oil-based lipid emulsion application could be subjected to induce PNALD disease model in hepatocytes which triggered ER, resulting in lipid droplet accumulation and ER stress. Besides, suppression of IRE1*α* attenuated the steatosis in PNALD cells, indicating that IRE1*α* pathway may have participated in the development and progression of PNALD.

## Data Availability

All the authors approved and confirmed the data availability, and all the data used to support the findings of this study are included within the article. Besides, the methods and materials are available from the corresponding author upon request.
